# Effect of polyols on thermostability of xylanase from a tropical isolate of *Aureobasidium pullulans* and its application in prebleaching of rice straw pulp

**DOI:** 10.1186/2193-1801-3-37

**Published:** 2014-01-18

**Authors:** Wichanee Bankeeree, Pongtharin Lotrakul, Sehanat Prasongsuk, Somporn Chaiareekij, Douglas E Eveleigh, Seung Wook Kim, Hunsa Punnapayak

**Affiliations:** Biological Sciences Program, Faculty of Science, Chulalongkorn University, Chulalongkorn, Bangkok, 10330 Thailand; Plant Biomass Utilization Research Unit, Department of Botany, Faculty of Science, Chulalongkorn University, Chulalongkorn, Bangkok, 10330 Thailand; Department of Imaging and Printing Technology, Faculty of Science, Chulalongkorn University, Chulalongkorn, Bangkok, 10330 Thailand; Department of Biochemistry and Microbiology, School of Environmental and Biological Sciences, Rutgers University, Rutgers, NJ 08901-8525 USA; Department of Chemical and Biological Engineering, Korea University, Seoul, 136-701 Republic of Korea

**Keywords:** Endoxylanase, Thermostability, Sorbitol, Paper production, Black yeast, Color variants, *Aureobasidium pullulans*

## Abstract

In an attempt to find a thermostable xylanase enzyme for potential application in the pretreatment prior to H_2_O_2_ bleaching of paper pulp for industry, an extracellular xylanase from *Aureobasidium pullulans* CBS 135684 was purified 17.3-fold to apparent homogeneity with a recovery yield of 13.7%. Its molecular mass was approximately 72 kDa as determined by SDS-PAGE. The optimal pH and temperature for activity of the purified enzyme were pH 6.0 and 70°C, respectively. The enzyme was relatively stable at 50°C, retaining more than half of its original activity after 3-h incubation. The thermostability of the enzyme was improved by the addition of 0.75 mM sorbitol prolonging the enzyme’s activity up to 10-fold at 70°C. When the potential of using the enzyme in pretreatment of rice straw pulp prior to bleaching was evaluated, the greatest efficiency was obtained in a mixture containing xylanase and sorbitol. Treatment of the rice straw pulp with xylanase prior to treatment with 10% (v/v) H_2_O_2_ and production of hand sheets increased the ISO sheet brightness by 13.5% and increased the tensile and tear strengths of the pulp by up to 1.16 and 1.71-fold, respectively, compared with pulps treated with H_2_O_2_ alone. The results suggested the potential application of the enzyme before the bleaching process of paper pulp when the maintenance of high temperature and enzyme stability are desirable.

## Introduction

Xylanolytic enzymes form a group involved in the hydrolysis of xylans and arabinoxylans, the most abundant hemicellulosic polymers in plant biomass. Within this group, endo-1,4-β-xylanase is of special interest for use in various industrial applications, especially biopulping and bleaching. Xylanases are used in the pretreatment of pulp prior to bleaching to increase the liberation of lignin through the hydrolysis of hemicellulose (Suurnäkki et al. 2004). They have been employed to reduce the subsequent use of toxic chemicals, such as chlorine and hydrogen peroxide (H_2_O_2_) (Beg et al. 2001). Although xylanases potentially offer a number of advantages over conventional chemical reagents, their application at an industrial scale remains limited. In the case of biobleaching, the incoming pulp for the enzyme-catalyzed process usually employs a high temperature (70–100°C) (Beg et al. 2001), at which commercial xylanases, such as Cartazyme®, Ecopulp X200®, and Resinase®, are not sufficiently stable (Viikari et al. 2002). Consequently, there is on-going search for more potent strains of xylanase producers, especially those that can produce thermostable enzymes with greater yields (Viikari et al. 2007).

Xylanolytic enzymes from *Aureobasidium pullulans*, generally known as black yeast, can be efficiently produced (Leathers 1986; Li et al. 1993; Ohta et al. 2010). The cellulase-free xylanases from *A. pullulans* produced efficiently (Leathers 1986; Ohta et al. 2010) are advantageous to the pulp and paper industry in that the hydrolysis of cellulose fibers is avoided resulting in greater yields of recovered pulp. From a number of xylanase-producing *A. pullulans* isolated from a range of different Thai habitats (Manitchotpisit et al. 2009), one strain (*A. pullulans* CBS 135684), a color variant, produced cellulase-free xylanase that was relatively thermostable. The stabilization of enzymes remains an important concern especially during thermal processing. The loss of enzyme activity throughout the elevated temperature ranges is related to changes of enzyme conformation (Cui et al. 2008; Fu et al. 2010). In order to prevent the conformational changes of the enzyme, addition of chemicals such as polyols to can promote numerous hydrogen bond or salt-bridge formation between amino acid residues which make the enzyme molecule more rigid, and therefore more resistant to the thermal unfolding (George et al. 2001; Costa et al. 2002). However, the selection of the appropriate additive depends on the nature of the enzyme. The objectives of this study were to (i) characterize the biochemical properties of purified thermally stable xylanase from an *A. pullulans* yeast strain, (ii) determine the effect of polyols on the thermostability of the enzyme, and (iii) investigate the potential application of the xylanase in the pulp bleaching of non-woody material. Rice straw was utilized as it is an alternative raw material that can be used for pulping with advantages of its porous fiber structure, greater concentration of holocellulose and less lignin levels (Rodrígueza et al. 2008). In addition, fibers from rice straw are shorter than softwood fibers which results in superior paper that can replace hardwood chemically treated pulps for printing and writing paper.

## Materials and methods

### Organism and culture conditions

*Aureobasidium pullulans* previously isolated, was maintained at the fungal culture collection of the Plant Biomass Utilization Research Unit, Department of Botany, Faculty of Science, Chulalongkorn University, Bangkok, Thailand (Manitchotpisit et al. 2009). It was deposited at the Centraalbureau voor Schimmelcultures, The Netherlands (CBS number 135684). The yeast was grown in yeast malt (YM) agar medium (Atlas 1993) at room temperature for 2 days and short-term stock cultures were stored at 4°C. For long-term storage, the strain was stored at -20°C in YM broth containing 20% (v/v) glycerol.

### Xylanase production

Seed culture was prepared by growing *A. pullulans* in basal medium (Leathers 1986) containing 1% (w/v) glucose at 30°C with 150-rpm agitation for 72 h. The inoculum was adjusted to 2.5 × 10^6^ cells. mL^-1^ and 100 μL was transferred into a 250-mL Erlenmeyer flask containing 100 mL of basal medium supplemented with 1% (w/v) agricultural wastes including wheat germ, wheat bran or corncob as the sole carbon source. The cultures were incubated at 30°C with 150-rpm agitation for 3 days. The cells were separated from the culture broth by centrifugation (18,000 × g, 20 min) at 4°C. The supernatant was used as the crude enzyme solution for further studies.

### Enzyme assay

Xylanase and cellulase activities were assayed (Bailey et al. 1992) at 70°C using 1% (w/v) beech wood xylan (Fluka, USA) and 0.5% (w/v) carboxymethyl cellulose with the degree of substitution of 0.65 (Sigma, USA) as the substrates in 50 mM acetate buffer (pH 6.0). The amount of released reducing sugars was determined by the 3,5-dinitrosalicylic acid (DNS) method (Miller 1959). One unit (U) of xylanase/cellulase was defined as the amount of enzyme required to release 1 μmol xylose/glucose equivalent per min under the optimal conditions. Results are reported as the mean value of three replicates.

### Xylanase purification

The extracellular xylanase was purified at 4°C. Culture supernatant (500 mL) from late log-phase (72 h) was concentrated ten-fold by ultrafiltration (10 kDa MW membrane cut-off, Amicon, Beverly, MA, USA) prior to precipitation using ammonium sulfate (50–80% saturation). After recovery (centrifugation 10,000 × g for 20 min), the pellet was dissolved in 20 mL 20 mM Tris–HCl buffer (pH 8.0) and dialyzed for 24 h against the same buffer. The enzyme solution (~28 mg total protein.mL^-1^) was applied (5 mL each run) to a pre-equilibrated anion exchange DEAE Sepharose FF (Sigma-Aldrich Co., USA) column. After washing the column with five-column-volumes of 20 mM Tris–HCl buffer (pH 8.0), elution was performed with a linear NaCl gradient (0 to 1.0 M over 50 mL) in the same buffer at a flow rate of 1.0 mL.min^-1^. Five-mL fractions were collected and analyzed for xylanase activity and protein content. The protein content was estimated by the Lowry method (Lowry et al. 1951), using bovine serum albumin (BSA) as the standard. Fractions with xylanase activity were pooled, dialyzed against 50 mM acetate buffer (pH 6), and then concentrated by ultrafiltration. Five mL of the concentrated enzyme solution (~4 mg total protein/mL) was applied to a 30 × 5 cm gel filtration column (Sephacryl S-100 HR, Sigma, USA) pre-equilibrated with 50 mM acetate buffer (pH 6.0). Elution was carried out at a flow rate of 0.5 mL.min^-1^, collecting 3 mL fractions that were assayed for xylanase activity and protein content as described above. The protein profiles of the collected fractions were determined by resolution through SDS-PAGE (12.5% (w/v) acrylamide resolving gel) as described (Laemmli 1970).

### Optimal conditions for xylanase activity

The optimal temperature for the purified xylanase activity was determined by incubating the reaction mixture (4.5 U.mL^-1^ enzyme and 1% w.v^-1^ xylan, total volume 5 ml) at various temperatures, ranging from 40–90°C and at different pHs. To determine the optimal pH for enzyme activity, 50 mM sodium acetate buffers of pH 3.0–6.0 and 50 mM phosphate buffers of pH 6.0–10.0 were used. The relative activity was calculated as the percentage of enzyme activity in comparison to the maximum activity.

### Effects of ions

To investigate the effect of ions on the enzyme activity, CaCl_2_, CuCl_2_, MgCl_2_, FeSO_4._7H_2_O, CoCl_2_, ZnCl_2_ and EDTA were separately added to the reaction mixture at two different final concentrations of 1 and 10 mM, respectively, prior to performing the enzyme assay under the optimum conditions. The relative activity was calculated as a percentage of enzyme activity without the addition of ion.

### Substrate specificity

The substrate specificity of the purified xylanase was tested with each of the followings; *viz*. beech wood xylan, oat spelt xylan, rice straw xylan, α-cellulose and carboxymethyl cellulose (CMC). Oat spelt and rice straw xylans were prepared according to the method of Höijea et al. (2005). The xylanase activity was assayed under the optimum conditions.

### Effect of temperature on enzyme stability and thermodynamic analysis

To determine the thermostability of the enzyme, it was incubated from 40-80°C, in the absence of substrate, and residual xylanase activity was determined every 30 min for up to 180 min. The data obtained from the thermal stability profile were used to analyze the thermodynamic parameters related to the xylanase activity. The half-life of the xylanase (*t*_1/2_, min^-1^) was determined from Eq. (1) (Sadana 1995):1

The *D*_t_ values (decimal reduction time or time required to inactivate 90% of the original enzyme activity at a constant temperature) were calculated from Eq. (2):2

The activation energy for xylanase denaturation (*E*_d_) was determined by an Arrhenius plot of the log denaturation rate constants (ln*k*_d_) versus the reciprocal of the absolute temperature in Kelvin (*T*) using Eq. (3) (Arrhenius 1889):3

where *R* is the gas constant (8.314 J.mol^-1^.K^-1^).

The changes in enthalpy (Δ*H*^o^, kJ.mol^-1^), free energy (Δ*G*^o^, kJ.mol^-1^) and entropy (Δ*S*^o^, J.mol^-1^.K^-1^) for the thermal denaturation of xylanase were determined using Eqs. (4)-(6) (Gummadi 2003)456

where *h* is the Planck constant (11.04 × 10^-36^ J.min) and *k*_B_ is the Boltzman constant (1.38 × 10^-23^ J.K^-1^).

### Effect of polyols on xylanase thermostability

In order to improve the thermal stability of the xylanase, polyols including ethylene glycol (2C), glycerol (3C), xylitol (5C), sorbitol (6C) and mannitol (6C) were added to separate enzyme solutions at 0.5 M final concentration prior to incubation at 70°C. Aliquots were withdrawn every 30 min, ice-cooled and then the residual xylanase enzyme activity was assayed under the optimal conditions. The stability of the enzyme was expressed as a percentage of residual activity (% RA) compared with activity of the initial enzyme activity (before incubation and no polyols). The polyol that most improved the thermostability was selected for further study over a range of concentration on the optimal (0.25–1.00 M) at 70°C.

### Pulp treatment and property determination

The rice straw was collected from a local rice field in Suphanburi province, Thailand. The pulping of rice straw was carried out with the soda process (Chaiarrekij et al. 2011) before being extensively washed with tap water to remove the alkali. The xylanase pretreatment (18.6 U crude xylanase.g^-1^ dry pulp) with or without sorbitol at a final concentration of 0.75 M, was performed in transparent plastic bags with 10% (w/v) rice straw pulp suspended in 50 mM sodium acetate buffer (pH 6.0). The reaction was performed at 70°C for 2 h (Viikari et al. 2007). Reducing sugars in the hydrolysates were determined by the DNS method. For H_2_O_2_ treatment, the enzyme-treated pulp (or the enzyme-untreated pulp as the control) was transferred to the H_2_O_2_ solution (10% (v/v) final concentration) and incubated for 1 h at the same temperature. The resultant bleached pulp was made into 60 g.m^-2^ hand sheets on a Rapid-Köthen sheet former (RK-2A KWT, PTI, Austria) according to the ISO Standard Method 5269–2. The brightness and opacity of the hand sheets were measured using an optical tester (Color Touch PC, Technidyne, U.S.A.), based on the ISO Standard Methods 2470 and 2471, respectively. The tensile and tear indexes were determined after tensile strength and tear resistance were measured using a tensile strength tester (Strograph E-S, Toyo Seiki, Japan) and a tear strength tester (Protear, Thwing-Albert, U.S.A.) according to TAPPI Standard Method T 494 om-01 and T414 om-04, respectively. Fiber morphology was also analyzed using a fiber quality analyzer (FQA LDA02, OpTest Equipment, Canada) according to the TAPPI Standard Method T271 om-12. Untreated pulp was used as the control.

### Data analysis

Statistical differences among the means of data were calculated using one-way analysis of variance (ANOVA) and Duncan’s Multiple Range Test (DMRT) or Student’s t-test (2 tailed) with the SPSS 17.0 software package (SPSS Inc., Chicago, U.S.A.). Differences at *P* < 0.05 were considered significant.

## Results and discussion

### Xylanase production

Xylanase production by *A. pullulans* CBS 135684 was investigated using several agricultural wastes with high hemicellulose contents as substrates including wheat germ, wheat bran and corncob (Goering and Van Soest 1970). The maximum xylanase production (4.10 U.mL^-1^) was observed using 1% (w/v) corncob for 3 days at room temperature (28 ± 2°C) (Table 1). Consequently, corncob was selected for further xylanase production. Even though the xylanase production from corncob was 1.76-fold lower than that obtained with beech wood xylan (7.23 U.mL^-1^), corncob was practically more attractive due to its lower cost (Benedetti et al. 2013). Similar approaches have been used with *Colletotrichum graminicola* (Zimbardi et al. 2013) and *Aspergillus nidulans* (Reis et al. 2003). However, the xylanases from these microbes included cellulase activity. None was detectable in the *A. pullulans* xylanase.

**Table 1:** **Xylanase activity produced by** ***A. pullulans*** **CBS 135684 cultivated in basal medium containing 1% (w/v) agricultural waste as the sole carbon source at room temperature (28 ± 3°C) for 3 days**

Agricultural wastes	Xylanase activity (U.mL^-1^)*
Wheat germ	3.25 ± 0.13^b^
Wheat bran	2.59 ± 0.08^a^
Corncob	4.10 ± 0.10^c^

*Mean ± one standard deviation derived from three replicates (*N* = 3). Different superscript letter (a,b,c) in the same column indicated the values were significantly different (ANOVA and DMRT, *P* < 0.05).

### Purification of the native extracellular xylanase from *A. pullulans* CBS 135684

**Figure 1:**
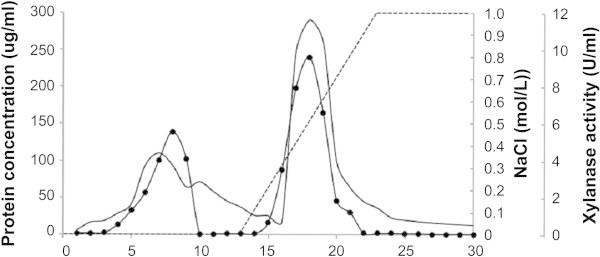
**Chromatographic separation of xylanases from** ***A. pullulans*** **CBS 135684 cultivated in basal medium containing 1% (w/v) corncob using DEAE-Sepharose [1.6 × 45 cm column - fast flow at 1.0 mL.min** ^**-1**^ **.** 5 ml fractions.]. ●, xylanase activity; (---), NaCl gradient; (-), protein concentration.

**Figure 2:**
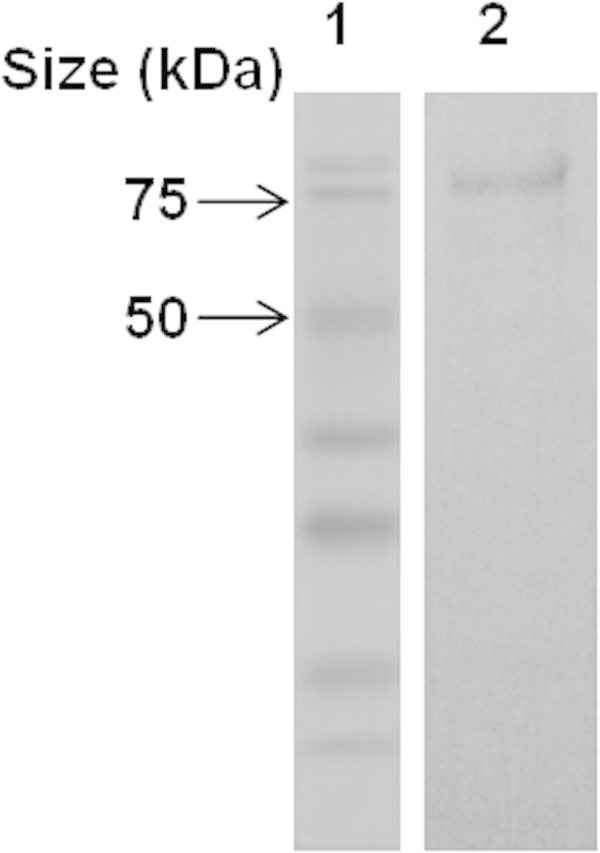
**SDS-PAGE analysis of the purified xylanase from** ***A. pullulans*** **CBS 135684 cultivated in basal medium containing 1% (w/v) corncob.** The enzyme (∼20 μg protein) was electrophoresed at pH 8.3 on a 12.5% acrylamide gel and stained with Coomassie Brilliant Blue R-250. Lane 1: MW standards; Lane 2: purified xylanase. The figure was a representative of repeated experiment.

The summary of the purification steps are shown in Table 2. DEAE-Sepharose column chromatography yielded two peaks of xylanase activity as isozymes (Figure 1). The result is similar to that reported for the color variant *A. pullulans* NRRL Y-2311-1 (Li et al. 1993) and *A. pullulans* ATCC 20504 (Tanaka et al. 2006) which secreted at least two isozymes of xylanase. The second peak, which represented the major portion (~60% total) of xylanase activity, was further fractionated using Sephacryl S-100 gel filtration chromatography to yield a single xylanase peak. The purified protein showed a single band of ~72 kDa on SDS-PAGE which coincided with the estimate by Sephacryl S-100 gel filtration chromatography (Figure 2). The overall level of recovery was approximately 13.7% with a 17.3-fold enrichment and a specific activity of 41.4 U.mg^-1^ protein.

**Table 2:** **Purification steps of xylanase isolated from** ***A. pullulans*** **CBS 135684 cultivated in basal medium containing 1% (w/v) corncob**

Purification steps	Total protein (mg)*	Total activity (U)^a,*^	Specific activity (U.mg^-1^)*	Purification (fold)*	Yield (%)*
Culture supernatant	2,030.00 ± 32.24	4,850.00 ± 36.42	2.39 ± 0.04	1.00	100.00
Ultrafiltration	1,376.00 ± 26.18	4,431.00 ± 27.31	3.22 ± 0.06	1.35	91.40
(NH_4_)_2_SO_4_ precipitation (50–80% saturation)	560.00 ± 1.24	2,440.00 ± 14.24	4.36 ± 0.02	1.81	50.30
DEAE-Sepharose	21.00 ± 1.04	834.00 ± 9.84	39.70 ± 1.76	16.60	17.20
Sephacryl S-100	16.00 ± 1.24	662.00 ± 10.46	41.40 ± 2.85	17.30	13.60

^a^1 U = the amount of enzyme required to release 1 μmol xylose equivalent per min under the assay conditions.

*Mean ± one standard deviation derived from three replicates (*N* = 3).

### Optimal conditions for xylanase activity

**Figure 3:**
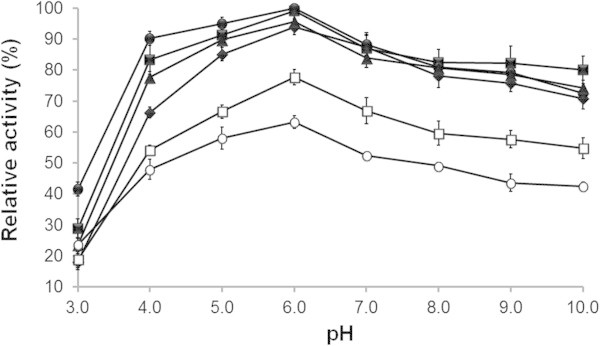
**Effect of pH and temperature on activity of the purified xylanase from** ***A. pullulans*** **CBS 135684 cultivated in basal medium containing 1% (w/v) corncob.** Reactions were conducted for 30 mins with 1% (w/v) beech wood xylan in 50 mM of sodium acetate buffers (pH 3.0–6.0) or phosphate buffers (pH 6.0–10.0) at various temperatures including 50°C (♦), 60°C (■), 70°C (•), 80°C (▲) and 90°C (ο). The relative activity was calculated as the percentage of enzyme activity assayed in 50 mM sodium acetate buffers (pH 6.0) at 70°C. Experiments were performed in triplicate (*N* = 3) and error bars indicated standard deviations.

The effects of pH and temperature on the activity of the purified xylanase are summarized in Figure 3. The purified xylanase was active over a broad pH range with a maximum activity at pH 6, and ≥ 80% and ≥ 70% maximal activity were observed at pH 4–8 and pH 4–10, respectively. The enzyme activity dramatically decreased at pH 3 (20% to 40% maximal activity at 50°C and 70°C, respectively). Conversely the xylanase activity was significantly increased (at each pH) as the incubation temperature was increased from 40°C to the optimal at 70°C (3.64 ± 0.12 to 4.60 ± 0.13 U.mL^-1^) at pH 6. A significantly lower xylanase activity was noted at 90°C (2.89 ± 0.05 U.mL^-1^).

The optimal pH (pH 6.0) and temperature (70°C) of the purified xylanase were similar to those of the crude enzyme (data not shown). Xylanases that are active in an alkaline environment and at high temperature are uncommon in yeasts (Techapun et al. 2002). Even among *A. pullulans* strains, previous reports on xylanase from *A. pullulans* (NRRL Y-2311-1 and ATCC 42023) showed that the enzymes were active at acidic pH (4.5-4.8) (Leathers 1989; Li et al. 1993; Vadi et al. 1996). Alkaline tolerant and thermotolerant xylanases have been reported in a number of bacteria, including *Bacillus* sp. (Zheng et al. 2000) and *Clostridium absonum* CFR-702 (Rani and Nand 2000). With its broad optimum pH and thermophilic properties, the xylanase from *A. pullulans* CBS 135684 may be used in several industrial applications not only for pulp bleaching but also for the bioconversion of lignocellulosic materials that is more optimally performed at a high pH and temperature (Parachin et al. 2009).

### Effects of metal ions

The effects of EDTA and six cations commonly found in pulp on the xylanase activity are summarized in Table 3. At 1 mM, none of the metal ions significantly affected the enzyme, except Ca^2+^. The presence of Ca^2+^ was equally stimulating at 1 and 10 mM, whilst Co^2+^ and Mg^2+^ significantly enhanced the xylanase activity only at 10 mM. The treatment of purified xylanase with EDTA did not significantly decrease the xylanase activity, suggesting that the divalent cation was not essential for xylanase activity. The presence of Zn^2+^ had no effect on xylanase activity which was different from a previous report that a purified xylanase from *A. pullulans* ATCC 42023 was inhibited by Zn^2+^ and Co^2+^ (Vadi et al. 1996). In contrast, Fe^2+^ and Cu^2+^ showed a dose-dependent inhibitory effect against the enzyme, which was significant at 10 mM. Inactivation of xylanases by Fe^2+^ and Cu^2+^ has been reported among other fungi including *Aspergillus usamii* (Zhou et al. 2009) and *Aspergillus awamori* VTCC-F312 (Do et al. 2012). These ions were suggested to react with the thiol groups, carboxyl groups and histidine residues in the enzymes and so destroy its active protein structure (Lama et al. 2004).

**Table 3:** **Effects of different cations with the purified xylanase from** ***A. pullulans*** **CBS 135684 cultivated in basal medium containing 1% (w/v) corncob**

Additive	Relative enzyme activity (%)* at an additive concentration of:	
	1 mM	10 mM
None	100.0 ± 2.0^abc, NS^	100.0 ± 1.0^C, NS^
Fe^2+^	99.9 ± 4.5^abc, 2^	74.1 ± 1.2^B, 1^
Mg^2+^	105.9 ± 3.0^c, 1^	111.7 ± 5.1^E, 2^
Ca^2+^	112.4 ± 1.5^d, NS^	112.4 ± 2.8^E, NS^
Cu^2+^	95.5 ± 2.2^a, 2^	29.9 ± 2.7^A, 1^
Co^2+^	103.4 ± 4.1^bc, 1^	106.1 ± 3.5^D, 2^
Zn^2+^	99.2 ± 2.5^ab, NS^	98.4 ± 2.4^C, NS^
EDTA	100.9 ± 5.3^abc, NS^	99.6 ± 1.2^C, NS^

The data were calculated from relative activity at different concentration of additives. The enzyme activity was assayed at 70°C for 30 min with 1% (w/v) beech wood xylan in 50 mM acetate buffer (pH 6.0).

*One hundred percent activity corresponded to the activity of the enriched xylanase under standard assay conditions (without additive). Mean ± one standard deviation derived from three replicates (*N* = 3). Different superscript letter (a,b,c,A,B,C,D,E) in the same column indicated the values were significantly different (ANOVA and DMRT, *P* < 0.05) and different superscript number in the same row (1,2) indicated the values were significantly different (Student’s t-test, *P* < 0.05).

NS = not significantly different.

### Substrate specificity

The activity of the purified xylanase on various substrates was determined. The enzyme showed high specificity towards different xylans. Among them, the highest activity was observed with 1% (w/v) beech wood xylan (4.10 ± 0.1 U.mL^-1^), followed by oat spelt xylan (3.87 ± 0.23 U.mL^-1^) and rice straw xylan (3.48 ± 0.12 U.mL^-1^), respectively. It might be due to the fact that the substitution of side chains in the cereal xylans was higher than that of the hardwood xylan (Voragen et al. 1992). In contrast, no activity was observed when using α-cellulose or CMC as substrate which indicated that the xylanase enzyme from *A. pullulans* CBS 135684 was cellulase-free (data not shown).

### Thermostability of the purified xylanase

**Figure 4:**
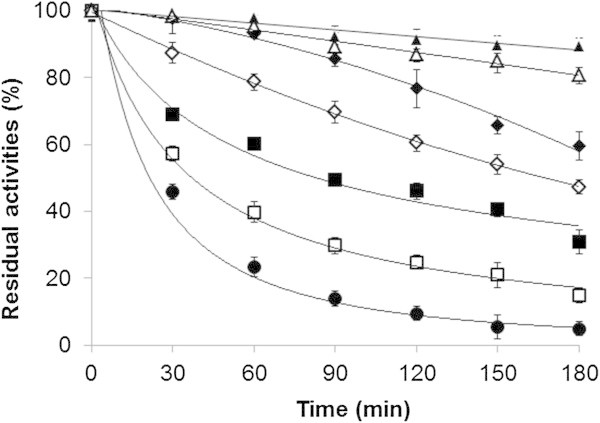
**Thermostability profile of the purified xylanase from** ***A. pullulans*** **CBS 135684 cultivated in basal medium containing 1% corncob.** The purified enzyme solubilized in 50 mM of sodium acetate buffers (pH 6.0) and separately incubated at 40°C (▲), 45°C (Δ), 50°C (♦), 55°C (⋄), 60°C (■), 65°C (□) and 70°C (•) prior to enzyme assay at standard pH and optimal temperature. The residual xylanase activity was calculated as the percentage of initial enzyme activity (before incubation). Experiments were performed in triplicate (*N* = 3) and error bars indicated standard deviations.

**Figure 5:**
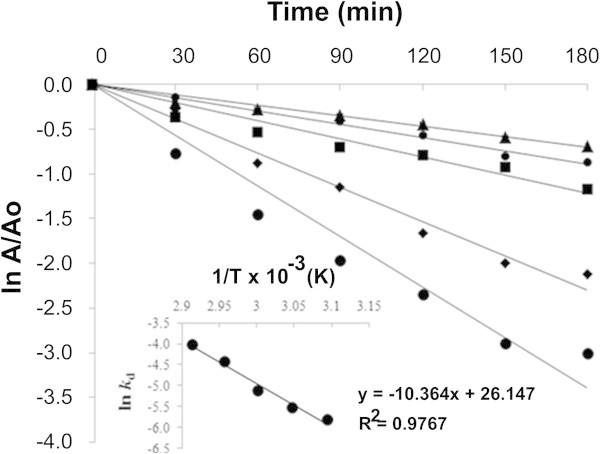
**First order thermal deactivation of the purified xylanase from** ***A. pullulans*** **CBS 135684 cultivated in basal medium containing 1% (w/v) corncob at 50°C (♦), 55°C (⋄), 60°C (■), 65°C (□) and 70°C (•).** Inset shows the Arrhenius plot for the calculation of activation energy (*E*_d_) for thermal denaturation. Experiments were performed in triplicate (*N* = 3) and error bars indicated standard deviations.

Stabilization of enzymes, especially during thermal processes, remains an important concern in modern biotechnology. The loss of enzyme activity during exposure to elevated temperatures is related to the significant changes in the enzyme conformation (Cui et al. 2008), and these can be estimated according to the Arrhenius equation and thermodynamic parameters (Marangoni 2003). In this study, the thermal stability of the xylanase from *A. pullulans* was assessed at 40-70°C at pH 6.0. The purified enzyme was found to be relatively stable at 40°C and 45°C with more than 80% of its original activity remaining after 3 h incubation (Figure 4). After incubation for 60 min at 50°C, 55°C, 60°C, 65°C or 70°C some 95%, 84%, 60%, 40% or 23% of the initial enzyme activity remained, respectively. However, the enzyme quickly lost its activity at 70°C and especially at 75°C and 80°C, it had < 20% residual activity after 30 min (data not shown). The semi-logarithmic plots of the residual enzyme activity versus incubation time were linear only at ranges between 50 to 70°C, with R^2^ > 0.97, indicating that the inactivation can be expressed as first order kinetics (Figure 5). The rate of enzyme deactivation (*k*_d_) evaluated from the slope of these plots; increased at higher temperatures (Table 4). The activation energy for irreversible inactivation (*E*_d_) of the xylanase was determined to be 86.1 kJ. mol^-1^ from the Arrhenius plot (Figure 5). Increasing the temperature decreased the half-life (*t*_1/2_) of xylanase. The enthalpy of activation of the thermal denaturation (Δ*H*) was decreased at 70°C, which clearly indicated that less energy was required for the thermal denaturation of the enzyme at a higher temperature. The Gibbs free energy (Δ*G*) for the thermal unfolding increased from 106.0-107.6 kJ.mol^-1^ with increasing temperature from 50–70°C. Although the unfolding of the enzyme structure was accompanied by an increase in the disorder or entropy of deactivation, the xylanase from *A. pullulans* CBS 135684 had negative entropy (ΔS), suggesting that the native form was in a more ordered state. The negative entropy change indicated aggregation of the xylanase in which a few inter- and/or intra-molecular bonds were formed, and thus the state of order of the system increased (Anema and McKenna 1996).

**Table 4:** **Thermodynamic parameters for the irreversible thermal inactivation of the purified xylanase from** ***A. pullulans*** **CBS 135684 cultivated in basal medium containing 1% (w/v) corncob**

Temperature (°C)	***k***_d_(min^-1^)	***t***_1/2_(min)	***D***_t_(min)	Δ***H*** ^o^(kJ.mol^-1^)	Δ***G*** ^o^(kJ.mol^-1^)	Δ***S*** ^o^(J.mol^-1^.K^-1^)
50	0.003	231	768	83.450	105.960	-0.070
55	0.004	173	576	83.400	106.850	-0.071
60	0.006	116	384	83.360	107.400	-0.072
65	0.012	58	192	83.320	107.100	-0.070
70	0.018	39	128	83.280	107.570	-0.071

The data were calculated from residual activity at different temperatures. The enzyme activity was assayed at 70°C for 30 min with 1% (w/v) beech wood xylan in 50 mM acetate buffer (pH 6.0).

Experiments were performed in triplicate (*N* = 3). *E*_d_ = 86.13 kJ.mol^-1^, Δ*H* ^o^ = Variations in enthalpy; Δ*G* ^o^ = Variations in free energy; Δ*S* ^o^ = Variations in entropy in free energy and Δ*S* ^o^ = Variations in entropy.

### Effect of polyols on the thermostability of the purified xylanase

**Figure 6:**
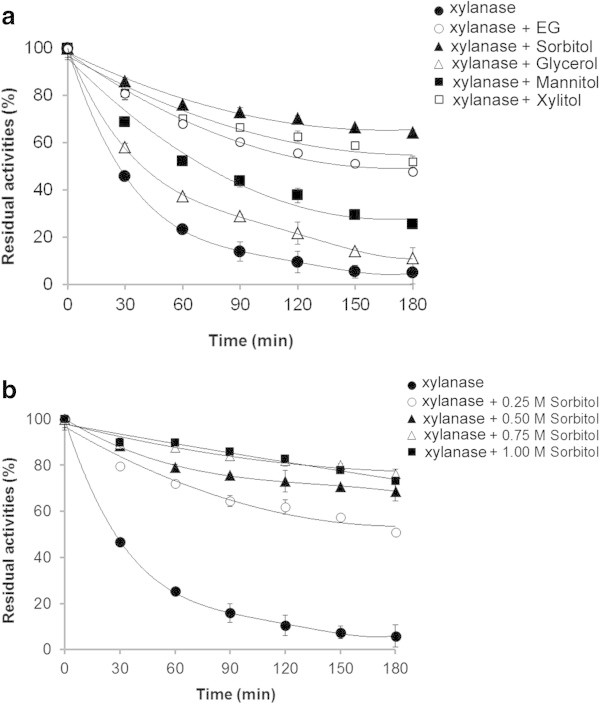
**The effect of polyols on thermostability of the purified xylanase from** ***A. pullulans*** **CBS 135684 cultivated in basal medium containing 1% corncob. (a)** The enzyme solutions were preincubated at 70°C without the presence of substrate for various times in the presence of glycerol (Δ), sorbitol (▲), mannitol (■), xylitol (□) and ethylene glycol (EG; ο) at a concentration of 0.5 M prior to enzyme assay at the optimal condition. **(b)** The enzyme solutions were incubated under the same condition as in **(a)** with the presence of sorbitol at concentrations of 0.25 M (♦), 0.50 M (■), 0.75 M (ο) and 1.00 M (▲) prior to enzyme assay at the optimal conditions. The residual xylanase activity was calculated as the percentage of initial enzyme activity (before incubation). Experiments were performed in triplicate (*N* = 3) and error bars indicated standard deviation.

In order to avoid thermal inactivation, the addition of small compounds to an enzyme solution can provide a practical means of increasing the enzyme stability by changing its microenvironment (Pal and Khanum 2010). For example, the addition of polyols improves the thermostability of enzymes from fungi (George et al. 2001; Kang-li and Da-nian 2013; Bourneow et al. 2012; Samborska et al. 2006; Lemos et al. 2000), including xylanases from *Trichoderma reesei* QM 9414 (Cobos and Estrada 2003) and *Aspergillus niger* DFR-5 (Pal and Khanum 2010). The selection of the appropriate polyol depends on the nature of the enzyme. In this study, the addition of each of five polyols showed a significant (*P* < 0.05) protection against thermal denaturation in comparison with the control (Figure 6a), but they differ in their degree of protection afforded. The highest protective effect was by sorbitol (6C, 6OH) which resulted in 64.2% retention of the original enzyme activity after 180 min at 70°C, some 14.5-fold higher than that for the enzyme alone. Xylitol (5C, 5OH) and ethylene glycol (2C, 2OH) gave the next highest protection, but respectively some 1.2- and 1.3-fold less effective than sorbitol. Mannitol (C6, 6OH) and glycerol (3C, 3OH) were far less effective protectants (2.3- and 5.1-fold less effective than sorbitol, respectively). Several studies have been suggested that the molecular size and number of hydroxyl groups per polyol molecule are play a crucial role in mediating the protection against thermal inactivation (Lemos et al. 2000). However, there is no clear pattern evident in this case. It has further been suggested that the protective role of polyols is due to their capability to form hydrogen bonds that support and stabilize the native conformation of the enzyme to make it more resistant to thermal unfolding (Cobos and Estrada 2003). In this study sorbitol was clearly the best thermoprotectant at 0.5 M. The effect of different sorbitol concentrations on the thermostability of the purified xylanase was also evaluated. Increasing the sorbitol concentration up to 0.75 M further improved the thermostability of the purified xylanase with ~80% of the original activity remaining after 180 min at 70°C (Figure 6b). At 1.0 M sorbitol, no significant further change in the xylanase activity was observed. While the effects of sorbitol on the thermostability of xylanase have been recognized in filamentous fungi including *Aspergillus awamori* (at 52°C) (Lemos et al. 2000) and bacteria including *Bacillus amyloliquefaciens* (at 80°C) (Breccia et al. 1998) and *Thermomonospora* sp. (at 80°C) (George et al. 2001), such effects on the thermostability of xylanase from yeast has not yet been reported.

### Prebleaching of rice straw pulp

Although the xylanase from *A. pullulans* CBS 135684 was relatively unstable at the temperature higher than 50°C (Figure 4), its stability could be improved by the addition of 0.75 M sorbitol (Figure 6). Half-life of the enzyme at 70°C was prolonged in the presence of sorbitol which was comparable to that at 50°C without sorbitol (data not shown). Moreover, in pulp and paper industry, the pulping and subsequent H_2_O_2_ bleaching processes are normally carried out around the temperature of 70–80°C (Sundara 1998). Fluctuated temperatures could cause fiber damage and pulp yield loss (Süss et al. 1996). Therefore, the condition of rice straw pulp pretreatment by xylanase in this study was set at 70°C. The effect of xylanase pretreatment on the pulp characteristics is summarized in Table 5. A significant (1.13-fold) increase in pulp brightness was observed with pretreatment of the rice straw pulp with either xylanase or xylanase plus 0.75 M sorbitol, compared to those of the pulp treated with H_2_O_2_ alone and the unbleached pulp. Increasing pulp brightness after xylanase bleaching was previously reported in bagasse pulp (Kulkarni and Rao 1996), and has been suggested to be caused by the selective hydrolysis of xylan on the fiber surfaces, which facilitates the subsequent H_2_O_2_ penetration into the fibers, leading to the release of lignin. The addition of 0.75 M sorbitol to the crude enzyme solution significantly increased the amount of reducing sugars released in the reaction mixture to 46.6 ± 3.05 μmole compared to 41.1 ± 1.3 μmole that was released from the pulp pretreated with the crude xylanase alone. The release of reducing sugars could have indicated the efficiency of xylan degradation. At the same time, xylanase pretreatment enhances the removal of transition metal ions, such as iron, manganese, magnesium and calcium, which cause the formation of colored metallic complexes in the carbohydrates (Kirk and Jeffries 1996).

**Table 5:** **The effect of xylanase pretreatment on the physical and optical properties of rice straw pulp**

Treatment	Brightness	Tensile index	Tear index	Fiber length	Fines content	Fiber curl	Fiber kink
	(ISO Units)*	(N.m g^-1^)*	(mN.m^2^g^-1^)*	(mm)*	(%)*	index*	index*
Untreated	39.5 ± 0.10^a^	39.8 ± 5.90	7.68 ± 0.50^a^	0.93 ± 0.05	49.3 ± 0.34^a^	0.07 ± 0.002^a^	1.48 ± 0.04^a^
H_2_O_2_	55.5 ± 1.00^b^	35.8 ± 7.00	6.56 ± 0.61^a^	1.01 ± 0.05	45.2 ± 0.14^b^	0.07 ± 0.003^a^	1.42 ± 0.03^b^
Xylanase + H_2_O_2_	62.8 ± 0.40^c^	42.4 ± 3.90	12.2 ± 0.75^b^	0.93 ± 0.05	40.6 ± 0.90^c^	0.09 ± 0.002^b^	1.59 ± 0.02^c^
Xylanase + Sorbitol + H_2_O_2_	63.0 ± 0.58^c^	46.3 ± 4.90	13.1 ± 1.30^b^	1.00 ± 0.16	39.7 ± 0.30^c^	0.10 ± 0.008^c^	1.65 ± 0.02^d^

All treatments were incubated in 50 mM of sodium acetate buffers (pH 6.0) at 70°C for 2 h for pretreatment and bleached at the same temperature for 1 h. The crude xylanase (18.6 U g^-1^ dry pulp) used in prebleaching experiment was obtained from *A. pullulans* CBS 135684 cultivated in basal medium containing 1% (w/v) corncob. Sorbitol (0.75 M) and H_2_O_2_ (10% (v/v)) were used in the respective experiment.

*Mean ± one standard deviation derived from three replicates (*N* = 3). Different superscript letter (a,b,c) in the same column indicated the values were significantly different (ANOVA and DMRT, *P* < 0.05).

From the observation of fiber morphology, no significant changes were noticed in the fiber length while the fines content was decreased by the xylanase-pretreatment of the pulp. The lower fines content led to the higher numeric average of fiber length and thus the paper strength was enhanced (Liu et al. 2012). In general, the strength of paper is influenced by the fiber morphology which can be adversely affected by the kink and curl of fibers (Page 1969). However, in this study, the paper strength, in terms of the tear and tensile indexes, increased along with the curl and kink indexes of the fibers after xylanase pretreatment. This may be due to the digested fibers becoming more flexible, thus the moderate curl of the fibers could enhance the interweaving between them which led to the strengthened bonding between the pulp fibers (Liu et al. 2012) These results clearly indicated that the crude xylanase from *A. pullulans* CBS 135684 had potential for treatment of rice straw before bleaching for paper manufacture since it significantly improved the paper brightness without compromising fiber quality.

## Conclusion

The extracellular xylanase produced by a Thai strain of *A. pullulans* CBS 135684 grown on corncob based media was cellulase-free and showed an enhanced activity at a high temperature (70°C). The purified enzyme was active over a broad pH range (> 70% activity at pH 4.0-10.0, and optimal at pH 6.0) which was uncommon among yeasts. The negative entropy change at all temperatures suggested that xylanase proceeds towards compaction during denaturation. The thermostability of the enzyme at high temperatures was improved by 0.75 mM sorbitol supplementation in the enzyme preparation. In the bleaching of rice straw fibers, enzyme pretreatment prior to H_2_O_2_ mediated bleaching significantly increased the fiber brightness and boosted the bleaching effect of H_2_O_2_. This new thermophilic xylanase from *A. pullulans* CBS 135684 has potential for use in the bleaching of rice straw fibers.
